# Stress and Food Consumption Relationship in Hypertensive Patients

**DOI:** 10.5935/abc.20190175

**Published:** 2019-09

**Authors:** Aline Lopes Dalmazo, Claudia Fetter, Silvia Goldmeier, Maria Claudia Irigoyen, Lucia Campos Pellanda, Eduardo Costa Duarte Barbosa, Thais Rodrigues Moreira, Denise Ruttke Dillenburg Osório

**Affiliations:** 1 Instituto de Cardiologia do Rio Grande do Sul - Laboratório de Investigação Clínica (LIC), Porto Alegre, RS - Brazil; 2 Universidade Federal de Ciências da Saúde de Porto Alegre, Porto Alegre, RS - Brazil; 3 Universidade FEEVALE, Novo Hamburgo, RS - Brazil

**Keywords:** Hypertension, Food Consumption, Stress, Physiological, Dietary Fats/metabolismo, Body Weights and Measures, Metabolism

## Abstract

**Background:**

Stress is a state of threat to the balance of the organism, which can cause biological and psychological changes. In hypertensive patients, stress can interfere with blood pressure levels, influence on food choices and neglect of the diet.

**Objective:**

This study aims to describe the relationship between stress and dietary intake of hypertensive patients.

**Methods:**

A transversal study was carried out at the Arterial Hypertension Clinic of the Cardiology Institute of Rio Grande do Sul, Brazil. The participants were aged ≥ 18 years and hypertensive. Blood pressure, food consumption and anthropometric measurements were collected. The variables related to stress were evaluated by the Lipp’s Stress Symptoms Inventory (LSSI) for adults. Significance level of 5% has been considered for all analyzed data.

**Results:**

The number of participants was 100. There was a higher prevalence of the female sex (67%), the mean age of the study population was 55.87 ± 12.55 years. Among the participants, 86% were classified in some of the stress phases, on which 57% were in the resistance phase. It was observed that there was no correlation between the presence of stress (as well as their actions), pressure levels and food consumption. The consumption of foods rich in lipids and individuals with a prevalence of psychological symptoms of stress displayed a significant association.

**Conclusions:**

Rich in fat dietary has been the first choice in patients with psychological symptoms of stress. Further studies regarding remodeled dietary intake and blood pressure levels in relation to the stress phases are suggested. These findings are important to contribute to the development of prevention and treatment strategies for cardiovascular diseases.

## Introduction

Stress is considered any force or experience that breaks the psychological homeostatic balance of an organism by activating a chain reactions cascade that increases blood flow through adrenaline release by adrenal glands and stimulates tachycardia, dilation of muscles and brain blood vessels and constricts blood vessels that supply the organs of digestion.^1^

According to the World Health Organization, stress affects more than 90% of the world's population and about 70% of Brazilians. Stress is a special situation where the development of considerable blood pressure changes may occur.^2,3^

Exposure to stress can lead to qualitative and quantitative changes in food consumption pattern,^4^ with higher and easier consumption of hyperpalatable foods. They have high-calorie density and are rich in fats and sugars, providing not only weight gain, but also contributing to the increase of chronic non-communicable diseases. Most of the time, stress promotes an increase in the consumption of this type of food, consequently decreasing the intake of fruits and vegetables.^5^ Emotional feeding may be related to behavioral and metabolic changes in the stress response.^4,6^

The effect of stress on a diet seems to modify the metabolism of various nutrients, such as B complex vitamins, C vitamin, calcium, magnesium, iron and zinc.^7,8^ Furthermore, when stress affects the patient, there is a tendency of neglecting the diet, aggravating pathological conditions by inadequate intake of nutrients. Among nutritional deficiencies, it stands out that mineral deficiencies are connected to a wide variety of metabolic dysfunctions.^8^

If the stress is continuous and intense, besides causing damage to the endocrine and immune system,^9^ it may lead to changes in lipid metabolism, blood pressure, heart rate, increased myocardial oxygen consumption and, as a consequence, reduction in peripheral vascular resistance.^6^ Progressively these problems lead to an increase in cardiovascular diseases.^10,11^ There is evidence that the major triggering factor for hypertension is precisely the stress exerted in moments of stress and anxiety that alter the entire hormonal and systemic configuration of the organism.^7^

Therefore, this study aims to evaluate the relationship between stress, food choices and food consumption in hypertensive patients.

## Methods

**Figure 1:**
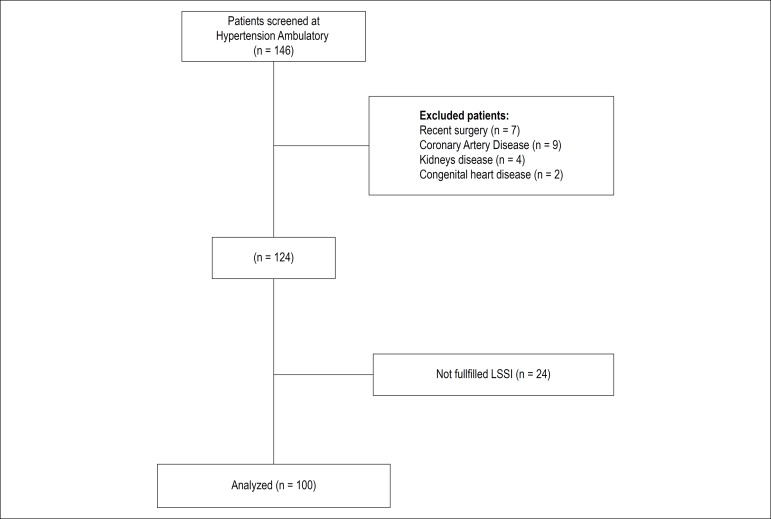
Flowchart of Participants. LSSI: Lipp stress symptoms inventory.

A cross-sectional study took place at Arterial Hypertension Clinic of the Cardiology Institute of Rio Grande do Sul, Brazil, between 2013 and 2015. Hypertensive patients of both genders, older than 18 years old, from Basic Health Units, with uncontrolled hypertension, in current use of antihypertensive medication and with an average diagnosis of hypertension of five years. Exclusion criteria were patients with secondary hypertension, congenital heart disease, surgical and acute myocardial infarction, as evidenced by the clinical file. The flowchart of patients invited to participate in the study is described in Figure 1.

In order to obtain the sample number to perform the study, the calculation was performed with 80% power, 95% confidence level and an expected correlation of r = 0.25 between the phases of stress and food consumption, totalizing a sample composed of 97 patients.^12^

Patients were invited to participate in the study after explaining the objectives, justification and methods that would be used in the data collection. After the acceptance, the patients signed two copies of the Free and Informed Consent Form, which was approved by the IC / FUC Research Ethics Committee, under No. 4843/13. It is important noticing that the study protocol complied with all ethical guidelines recommended by the National Health Council.

The data collection was conducted by training professionals in the Cardiology Institute settings during a single scheduled consultation in the multidisciplinary outpatient clinic of Systemic Arterial Hypertension. Variables of age, gender, blood pressure, anthropometry, food consumption and stress symptoms were collected.

The nurse team performed measures of blood pressure in accordance with the VII Brazilian Guidelines for Hypertension.^2^ Anthropometric measurements were taken by the nutritionist. Weight and height variables were collected using an anthropometric scale (Welmy®), with a capacity of 200 kg and with anthropometric ruler coupled up to 2 meters. For weighing, all patients were instructed to be barefoot to remove objects from their pockets, watches and excess clothing. The patient was placed in the center of the platform, with arms extended along the body. For height measurement, the patient remained barefoot and standing upright, with his head held high, arms hanging at his side, heels and back against the vertical plane of the rod. In sequence, the patient was instructed not to shrink when the stem was placed on the head, the support of which remained on the scalp, avoiding contact only on the hair. Weight and height data were used to calculate body mass index (BMI) and the following cutoff points were used for adults: Low weight < 18.5 kg/m^2^; Eutrophic 18.5 - 24.9 kg/m^2^; Overweight 25 - 29.9 kg/m^2^ and Obesity ≥ 30 kg/m^2^; ^3^ and for elderly: Low weight: < 22 kg/m^2^; Eutrophic: 22 - 27 kg/m^2^ and Excess weight:> 27 kg/m^2^ according to Lipschitz (1994).^13^

For the qualitative evaluation of food consumption, food frequency questionnaire adapted from Ribeiro et al.,^14^ was applied in order to analyze the frequency of food consumption. The interpretation of this questionnaire was carried out through the stratification of the consumed food groups, being evaluated the following groups: ultra-processed foods, *in natura* foods, rich in carbohydrates, proteins and lipids.

Lipp's stress symptoms inventory (LSSI) for adults was applied by a psychologist to evaluate the symptoms of stress. This inventory is an objective measure of stress symptomatology in individuals over 15 years of age. LSSI is composed of 37 somatic and 19 psychological items whose symptoms, if repeated, differ only in intensity and severity. This instrument is divided into 3 sets: the 1st with 15 items refers to the physical or psychological symptoms that the patient has experienced in the last 24 hours; the second, composed of ten physical and five psychological symptoms, and is related to the symptoms experienced in the previous week; the third, with 12 physical and 11 psychological symptoms, refer to the situation of the previous month.^15^

### Statistical analysis

Data tabulation was performed in the Microsoft Excel 2013 for Windows. The statistical analysis was performed using the Statistical Package for Social Sciences (SPSS), version 22.0. Variables with normal distribution were described in mean ± standard deviation. Variables with normal distribution were described in mean ± standard deviation and variables with asymmetric distributions such as median and interquartile range (25th and 75th percentile).

In order to correlate dietary intake of different food groups (carbohydrates, lipids, proteins, *in natura* and ultra-processed foods) with the stress phases, the Spearman correlation coefficient was used.

For the comparison of continuous variables concerning the presence or absence of stress, the Mann-Whitney test (food intake) and Student's t-test (blood pressure) were used for independent samples. In regards to the types of stress symptoms (physical/psychological/mixed) the tests used were Kruskal Wallis (food consumption) and ANOVA One-way (blood pressure). Significance level of 5% (p < 0.05) was considered.

## Results

The sample consisted of 100 patients with a mean age of 55.87 ± 12.55 years and 67% (n = 67) were females. The mean values of blood pressure were 182.38 ± 28.01 mmHg for systolic and 94.95 ± 12.42 mmHg for diastolic. Regarding the stress variable, 86% (n = 86) of the participants were included in some of the phases, of which 57% were in the resistance phase (Table 1).

**Table 1:** Characterization of participants

CARACTERÍSTICAS		n	%
**Gender**			
Women		67	67%
Men		33	33%
Age (mean ± standard deviation)		55.87 ±12.55	
(minimum- maximum)		(19 a 80)	
**BLOOD PRESSURE**			
SBP		182.38 ± 28.01	
DBP		94.95 ± 12.42	
**ESTRESSE – LSSI**			
Alert		2	2%
Resistence		57	57%
Almost exhaustion		11	11%
Exhaustion		16	16%
No stress		14	14%
**Nutritional State – BMI**			
Eutrophic		31	31%
Overweight		69	69%

LSSI: Lipp Stress Symptom Inventory; SBP: systolic blood pressure; DBP: diastolic blood pressure; BMI: body mass index.

No correlation was observed between the different stages of stress, blood pressure and food consumption (Table 2).

**Table 2:** Correlation of the Stress phases with food consumption and blood pressure

		LSSI Phases		SBP		DBP
SBP		0.023 (p = 0.821)		-		-
DBP		0.134 (p = 0.185)		0.449 (p > 0.01)		-
Ultraprocessed		-0.059 (p = 0.563)		-0.003 (p = 0.980)		0.070 (p = 0.490)
Carbohidrates		0.008 (p = 0.938)		-0.074 (p = 0.467)		-0.115 (p = 0.253)
Proteins		-0.154 (p = 0.125)		-0.044 (p = 0.663)		-0.064 (p = 0.524)
In natura		-0.002 (p = 0.987)		-0.113 (p = 0.262)		-0.083 (p = 0.413)
Lipids		0.160 (p = 0.313)		-0.193 (p = 0.220)		0.003 (p = 0.987)

Spearman Correlation Coefficient - correlation between stress phases (alert, resistance, near exhaustion and exhaustion) with food consumption (carbohydrates, proteins, lipids, ultra-processed and in natura foods) p < 0.05. LSSI: Lipp Stress Symptom Inventory; SBP: systolic blood pressure; DBP: diastolic blood pressure.

Table 3 shows the comparison of the food consumption profile with the presence or absence of stress and blood pressure.

**Table 3:** Food consumption and blood pressure according to the classification of present or absent Stress

		LSSI				p
		Absent		Present		
Ultraprocessed		3 (1.1; 3.5)		2.4 (0.7; 3.4)		0.295
Carbohydrates		0.3 (0.2; 0.9)		1.1 (0.3; 2.3)		0.099
Proteíns		2.5 (2; 3.2)		2.4 (1.7; 3.1)		0.522
*In natura*		3.5 (2.7; 3.9)		3.3 (2.7; 4.2)		0.761
Lipids		4.3 (3.5; 6.5)		7 (3.6; 7)		0.367
SBP (mmHg)		168.07 ± 27.31		174.36 ± 26.58		0.416
DBP (mmHg)		90.93 ± 15.83		93.51 ± 15.56		0.568

Mann-Whitney and SBP (systolic blood pressure) and PAD (diastolic blood pressure) were used for food consumption (consumption on days of the week). Test T. LSSI: Lipp Stress Symptom Inventory for Adult.

Among the food groups investigated, there was a significant association with foods rich in lipids and psychological symptoms of stress, according to Table 4.

**Table 4:** Comparison of frequency of food consumption and blood pressure in relation to the types of stress symptoms

Outcomes	LSSI			p
	Physical	Psychological	Mixed	
Ultraprocessed	1.9 (0.5; 2.8)	3.4 (1.3; 4.9)	2.4 (0.8; 2.9)	0.065^#^
Carbohidrates	1.1 (0.3; 2.5)	1.1 (0.3; 1.7)	1.4 (0.2; 2.7)	0.573^#^
Proteins	2.3 (1.6; 3)	2.4 (1.7; 3.2)	2.4 (1.6; 3.2)	0.848^#^
*In natura*	3.3 (2.6; 4)	3.6 (2.9; 4.2)	3.1 (2.6; 4.2)	0.608^#^
Lipids	5 (3.5; 7)	7 (7; 7)	5 (3.3; 7)	0.026^#^
SBP	170.66 ± 27.1	182.38 ± 28.01	177.92 ± 23.82	0.226*
DBP	91.92 ± 15.74	94.95 ± 12.42	100.15 ± 18.81	0.226*

# Kruskal Wallis Test – Values presented as mediana/interquartile interval.

* ANOVA – Values presented as Mean (M) ± Standard Deviation (SD).

LSSI: Lipp Stress Symptom Inventory; SBP: systolic blood pressure; DBP: diastolic blood pressure.

## Discussion

This study aimed to describe the relationship between stress and food consumption in hypertensive patients. Analyzing the consumption of foods rich in lipids in individuals with stress and predominance of psychological symptoms, we found a significant association between the variables.

Regarding the predominance of women, it is believed that a more significant number of them seek care assistance and show higher health concerns when compared to the men.^16^

In a review of stress-induced eating, Greeno and Wing^17^ have shown that different stress stimuli cause different reactions, taking into account the individuality of the patients, but stress can affect the quality of food choice.

Nguyen et al.^18^ demonstrated in his study on stress-induced eating with 517 students that perceived stress was a significant correlate of "emotional eating" and also added in their results that this factor is independent of BMI, suggesting there is no relation between stress-induced eating; and people who are overweight and obese.

In the present study, we did not observe a significant positive relationship between food consumption in different stages of stress with the presence or absence of stress. The divergences found in this study concerning those mentioned above can be attributed to the diversity of the analyzed variables, which suggests a more detailed investigation, as it is well documented by Sousa et al.^19^ that the stimuli and physiological responses differ in each phase of the stress.^19^

Regarding stress, in the resistance phase (57%), it is in agreement with the results obtained by Wottrich et al.,^20^ in which the predominance of the individuals evaluated was predominant in the endurance phase. These findings are in agreement with data from the study described by Malagris^21^ and Rosseti ^22^ whose results were similar in their research.

Lipp et al.^23^ also emphasizes that the resistance phase is associated with excessive fatigue, memory problems and doubts about oneself, which can significantly compromise the individual's quality of life.

Pecoraro et al.^24^ and Zellner et al.^25^ stated that in order to minimize stress symptoms, it is common to eat tasty foods, mostly high in fat, as a form of comfort and "self-medication". In another study with adolescents conducted in London, it was found that a high degree of perceived stress was related to high intakes of fat and large amounts of unhealthy meals.^5^ In this study, a positive association between the consumption of rich foods in lipids in patients classified with some level of stress, with a predominance of psychological symptoms.^5^

It is important to point out that fat in the food promotes greater palatability being also more caloric. The fact that psychological symptoms are predominant may reveal that the individuals were worried, with low self-esteem and irritated, therefore, with their psychological conditions compromised, seeking for some compensation and well-being in food. It is known that emotions can determine food choices and preferences and foods are associated with the emotional context in which they are usually consumed.^26,27^

Considering that primary prevention of blood pressure elevation can be achieved by controlling risk factors including changes in lifestyle, multimodal interventions are indicated to integrate education on healthy lifestyle and medical resources, physical activity, stress management and counseling on psychosocial risk factors.^28^

The present study presented some limitations, we highlight the application of the questionnaire of the frequency of food consumption. This method requires greater precision to remember the foods consumed in the different frequencies evaluated, which could potentially be considered a memory bias. However, among the types of validated food consumption protocols, this is considered to be more reliable and representative of food when compared to the 24-hour food recall or food diary. Another difficulty observed was the small number of scientific studies on the subject, which made it difficult to deepen the discussion of the data.

In the population of hypertensive patients, it is necessary to explore team strategies for better management of stress, as well as to prescribe a reduction in the intake of fatty foods and to accompany them, which will imply the effectiveness of disease control, risk control related to comorbidities and better quality of life.

As future work, we intend to evaluate the relationship between altered dietary intake and blood pressure levels regarding specific stages of stress. Indeed, the analysis of this study shows itself useful for hypothesis assessment for future researchers.

## Conclusion

Changes in dietary choices were evidenced by higher consumption of high-fat foods in individuals with a prevalence of psychological symptoms. However, more studies are needed the alteration of food consumption and blood pressure levels in relation to certain stages of stress. Because hypertension is a multifactorial disease, it requires a multi-area treatment approach to achieve better results. These findings are essential to contribute to the development of new strategies for the prevention and treatment of diseases, thus minimizing the risk factors for the progression of cardiovascular diseases.
